# On Tubercular Degeneration as the Most Frequent Cause of Acute Hydrocephalus

**Published:** 1841-07

**Authors:** 


ART. XI.—*Ueber Tuberkulose als die gewohnlichste Ursache des Hydrocephalus Acutus.* Von Dr. F. SCHWENINGER.—*Regensburg*, 1839. 8vo, pp. 88.

*On Tubercular Degeneration as the most frequent cause of acute Hydrocephalus.* By Dr. SCHWENINGER.—*Ratisbon*, 1839.

IN this unpretending but valuable pamphlet Dr. Schweninger has embodied the results of the post-mortem examinations of all the fatal cases of hydrocephalus which occurred at Ratisbon between the years 1835 and 1839. The observations are twenty in number, and the morbid appearances are detailed with much care and minuteness; but unfortunately the author has not been able to give equally accurate descriptions of the course of the disease, since the patients seldom came under his notice during their lifetime.

In every instance tubercles were found in one or more internal organs: in four cases they existed in all the visceral cavities; in ten in the chest and abdomen; in five in the chest alone; and in one only they were confined to the abdomen. The different organs may be arranged in the following order according to the frequency of the tubercular deposit in them: bronchial glands, 17 times; lungs, 15; spleen, 10; mesenteric glands, 7; liver, 7; brain, 4; pleura, peritoneum, stomach, and kidneys, once in each. In five instances, too, the author met with that granular condition of the arachnoid membrane to which the name of meningitis tuberculosa has been applied by some pathologists. From this commonly received opinion, however, Dr. Schweninger dissents, apparently without being aware that the same view of the subject had been taken by Rouchoux in an article published in the Journal Hebdomadaire for 1835. He regards this deposit as being, like the so-called glandulæ pacchioni, a simple exudation analogous to those granular false membranes which are sometimes found on the pleura or peritoneum in cases where no organs present the slightest trace of tubercle.

From his observations he deduces the following results: 1st. That the meningitis which accompanies hydrocephalus does not differ in its anatomical characters in any important respect from meningitis when simple or when complicated with other morbid conditions. 2d. That the meningitis accompanying hydrocephalus does, however, differ from simple meningitis or from that associated with other morbid conditions, in being combined with other simultaneous pathological changes. 3d. That meningitis, though a frequent complication of hydrocephalus, is not the cause of the disease. 4th. This meningitis complicating hydrocephalus is itself only the result of the more or less general deposit of tubercle. 5th. The hydrocephalus is in all the above cases the result of tubercle, and there is great probability in the supposition that the greater number of cases of hydrocephalus may be ascribed to this cause.

In conclusion he endeavours to show that there is nothing in the supposition that infantile hydrocephalus consists in effusion consequent on tubercular degeneration, contrary to the analogies of tubercular disease in general. In that malady nothing is more common than to find abnormal collections of fluid the product either of secretion or of excretion. Œdema of the feet and general anasarca are both by no means unusual in the advanced stages of phthisis. A much more abundant collection of fluid in the ventricles and under the arachnoid is met with in phthisical subjects, than in those who have died of most other diseases. hydropericardium and œdema of the lungs, membranes of the intestines, &c., are common in tubercular patients. The copious diarrhœas and profuse perspirations of phthisical patients are instances of preternatural excretions of fluid, incidental to that disease. The occasional occurrence of serous effusion into the ventricles after the suspension of the diarrhœa in these cases is another circumstance which speaks for the existence of a common cause for both conditions, and is regarded by the author as favouring his supposition of the frequent production of hydrocephalus as a consequence of preexisting tubercular disease.

